# Connecting Families to Food Resources amid the COVID-19 Pandemic: A Cross-Sectional Survey of Early Care and Education Providers in Two U.S. States

**DOI:** 10.3390/nu13093137

**Published:** 2021-09-09

**Authors:** Lacy Stephens, Caroline Rains, Sara E. Benjamin-Neelon

**Affiliations:** 1 National Farm to School Network, P.M.B. #104, 8770 West Bryn Mawr Ave, Suite 1300, Chicago, IL 60631, USA; 2 Research Triangle Institute International, 3040 East Cornwallis Road, Research Triangle Park, NC 27709, USA; crains@rti.org; 3 Department of Health, Behavior and Society, Johns Hopkins University, 615 North Wolfe Street, Baltimore, MD 21205, USA; sara.neelon@jhu.edu

**Keywords:** CACFP, COVID-19, early care and education, food program

## Abstract

Early care and education (ECE) settings are important avenues for reaching young children and their families with food and nutrition resources, including through the U.S. federally funded Child and Adult Care Food Program (CACFP). Researchers conducted a cross-sectional survey of ECE providers in two U.S. states in November 2020 to identify approaches used to connect families with food and nutrition resources amid the COVID-19 pandemic. Logistic regression models were used to estimate odds of sites reporting no approaches and adjusted Poisson models were used to estimate the incidence rate ratio of the mean number of approaches, comparing sites that participate in CACFP to those that did not. A total of 589 ECE sites provided responses. Of those, 43% (*n* = 255) participated in CACFP. CACFP participating sites were more likely to report using any approaches to connecting families to food resources and significantly more likely to report offering “grab and go” meals, providing meal delivery, distributing food boxes to families, and recommending community food resources than non-CACFP sites. This study suggests that CACFP sites may have greater capacity to connect families to food resources amid emergencies than non-CACFP participating sites.

## 1. Introduction

Early care and education (ECE) sites, including child care centers, family child care homes, Head Starts, state or private preschools, nurseries, and childminders, are important settings for impacting nutrition and health behaviors in young children and reaching families with nutrition information [1,2,3,4]. Across “economically advanced” countries, 25% of children under the age of 3 years and 80% of children ages 3–6 are in some form of ECE [5]. In the United States (U.S.), 60% of children under the age of 5 years regularly spend time in non-parental care, and those children spend an average of approximately 25 h per week in care [6]. The Academy of Nutrition and Dietetics recommends that children in full-time care consume half to one-third of their daily calories in care, highlighting the importance of ECE sites as access point for healthy food for young children [7].

The Child and Adult Care Food Program (CACFP) is a federally funded, state-administered child nutrition program of the U.S. Department of Agriculture (USDA) that provides reimbursement to child care providers for eligible meals and snacks served to children in care [8]. Reimbursable meals and snacks must meet specific nutrition standards and meal patterns, which were most recently updated in 2010 U.S. legislation the Healthy Hunger Free Kids Act and implemented in 2017 [9]. CACFP participation is associated with more nutritious meals and better adherence to nutrition recommendations [10,11,12,13,14], greater capacity to connect with families [15], and reduced risk for household food insecurity for participating families [16]. However, while food offered at CACFP participating sites may be more nutritious, CACFP participation may not be associated with increased intake of nutritious foods by children [13]. Barriers to serving healthy foods and meeting healthy foods standard in ECE settings include cost of foods, child acceptance of foods, and provider time constraints for food purchasing and preparation [17,18,19]. One study suggests that CACFP participation may be associated with fewer ECE provider reported barriers to serving healthy foods [20].

Starting in the spring of 2020, the COVID-19 pandemic wreaked havoc on health and economic systems across the world, with reverberating impacts on families and children. Food insecurity in the U.S., particularly amongst families with children, increased dramatically. In November 2020, 28% of families with children reported being food insecure, nearly a threefold increase from pre-pandemic rates [21,22]. In the decade prior to the COVID-19 pandemic, household food insecurity rates in the U.S. had been steadily declining to a rate of approximately 10.5% in 2019 [23]. Globally, food insecurity has risen in recent years and the COVID-19 pandemic caused dramatic increases in acute food insecurity worldwide [24]. Families with children, particularly low-income families, were more likely to have lost income during the COVID-19 pandemic [21]. The Urban Institute reported 4 out of 10 parents with children under 6 lost employment and/or income in 2020 [25].

ECE programs and providers were severely impacted by the COVID-19 pandemic. Over half of ECE programs in the U.S. shut down at some point during the first 4 months of the pandemic and 18% remained closed as of November 2020 [26,27]. ECE site closures compounded family financial challenges and food insecurity by reducing already limited access to affordable child care, limiting parents’ ability to work outside the home, and reducing access to meals and snacks served in ECE [21,28]. Even ECE sites that stayed open had limited capacity and resources to provide meals and nutrition resources [29].

As the impacts of the COVID-19 pandemic continue, the potential long-term impacts on child health are unclear. Early studies in school age children showed that the COVID-19 pandemic may have contributed to unhealthier eating habits, reduced physical activity, and increased screen time, all factors that may contribute to unhealthy weight gain [30,31]. ECE-based health and nutrition interventions may be increasingly important to mitigate potential long-term impacts of the COVID-19 pandemic on children’s and families’ access to sufficient and health-supporting food. Though initial data have been collected on CACFP participants’ immediate responses and operational challenges due to the COVID-19 pandemic [32], the authors are not aware of any study yet to look at differences in food and nutrition response based on CACFP participation.

The purpose of this research was to assess ECE site participation in initiatives that connect children and families to food and nutrition resources amid the COVID-19 pandemic, comparing sites that did and did not participate in CACFP. Based on existing literature, the authors hypothesize that CACFP sites were more likely to participate in food- and nutrition-related initiatives than non-CACFP participating sites.

## 2. Materials and Methods

### 2.1. Study Design and Sample

Researchers conducted a cross-sectional survey of ECE providers from two U.S. states, Arizona and Pennsylvania, in the fall (November) of 2020. The target states were selected based on researcher relationships to these locales and access to complete lists of licensed ECE provider emails in the states. These lists were obtained from the respective states’ ECE licensing agency websites. Researchers emailed a recruitment letter with survey link and consent information via Qualtrics to a total of 8171 recipients (2190 from Arizona and 5981 from Pennsylvania). Removing failed, bounced, and SPAM emails, a total of 7507 emails were delivered (1952 from Arizona and 5555 from Pennsylvania). The survey was open for responses for four weeks and two reminder emails were sent to non-respondents during the survey period. Participants who completed the survey were entered to win a random drawing for a $100 Amazon gift card. The survey tools and procedures were approved by Johns Hopkins Bloomberg School of Public Health Institutional Review Board.

### 2.2. Survey

The survey was designed to explore perceived impacts of the COVID-19 pandemic on ECE sites, including food- and nutrition-related activities and initiatives. The survey took approximately 10 min to complete and consisted of a total of 21 questions. The survey included 7 questions related to general program information, 5 questions on COVID-19 pandemic-related program model changes and perceived impact, 3 questions specific to CACFP and meal service, and 7 questions on activities, interests, and barriers related to serving and teaching about local food. Survey questions were primarily multiple choice with one Likert scale response and one open ended question. Questions were drawn from previous surveys related to food initiatives in ECE sites and the food-related COVID-19 pandemic response of ECE sites [32,33].

### 2.3. Analysis

To summarize descriptive characteristics of ECE sites, researchers calculated frequencies and percentages for categorical variables and means and standard deviations for continuous variables. The primary study outcome was reporting no approaches to connecting families to food and nutrition resources. Secondary study outcomes included mean number of approaches reported among sites reporting at least one approach and specific approaches used (offered “grab and go” meals, offered meal delivery, distributed food boxes, recommended community food resources, provided food from an on-site garden). Descriptive statistics were used to assess ECE site approaches to connecting families to food and nutrition resources amid the COVID-19 pandemic (March–November 2020). Unadjusted and adjusted logistic regression models were used to estimate odds of sites reporting no approaches to connecting children and families to food and nutrition resources since the onset of the COVID-19 pandemic and odds of sites reporting using specific approaches to connecting children and families to food and nutrition resources, comparing sites that participated in CACFP to those that did not. Unadjusted and adjusted Poisson models were also used to estimate the incidence rate ratio of the mean number of approaches used to connect families to food and nutrition resources among sites reporting at least one approach, comparing sites that participate in CACFP to those that do not. Sites reporting no approaches were removed from analysis comparing specific approaches and total number of approaches by CACFP status in order to assess differences only across those who reported approaches. This approach allows for more straightforward interpretation by key audiences (early childhood stakeholders and policy makers) and direct comparison to existing literature that uses the same analytical approach [20]. Models were adjusted for child enrollment (number of children enrolled in November 2020) and program type (family child care home, child care center/Head Start, or preschool); these potential confounders were selected *a priori* based on evidence that these factors differ between CACFP and non-CACFP participating sites [34]. State was included as an indicator in both adjusted and unadjusted models. Exploratory analysis was conducted to assess differences in approaches to connecting families to food and nutrition resources by site closure duration.

## 3. Results

### 3.1. Respondent Site Characteristics

A total of 589 ECE sites provided usable responses to the survey. This is a response rate of 8%. Of those respondents, 43% (*n* = 255) participated in CACFP and 57% (*n* = 334) did not participate in CACFP (Table 1). The distribution of program type across all respondents was 22% (*n* = 127) family or group child care home, 61% (*n* = 359) child care centers, 1% (*n* = 8) Head Start or Early Head Start, and 16% (*n* = 91) preschool programs (including state funded, private, and programs through K-12 schools). There were some differences in program type distribution between CACFP participating and non-participating sites, with more CACFP sites being family or group child care homes (33%, *n* = 83) than non-CACFP participating sites (13%, *n* = 44) and fewer CACFP sites being preschool programs (5%, *n* = 13) than non-CACFP participating sites (23%, *n* = 78). Approximately 21% of responding sites were from Arizona (*n* = 124) and 79% were from Pennsylvania (*n* = 465).

Sites reported a mean number of 67 (SD = 90) children enrolled in February 2020 (pre-COVID-19 enrollment) and 44 (SD = 55) children enrolled in November 2020. Mean enrollment in both February 2020 and November 2020 was similar across CACFP participating and non-participating sites. Mean site enrollment decreased 34% between February and November across all sites.

### 3.2. Impacts of the COVID-19 Pandemic on ECE Sites Operations

Respondents were asked to report how severely, overall, the COVID-19 crisis impacted their organizations. More than half of sites (56%, *n* = 331) reported significant impacts from the COVID-19 pandemic. Most of the remaining sites reported moderate impacts (42%, *n* = 246), with just 2% (*n* = 9) reporting no impacts (Table 2). The reported impact of the COVID-19 pandemic on sites did not vary significantly across CACFP and non-CACFP participating sites. In regard to COVID-19 pandemic-related site closures, most sites reported closure between one month and less than three months (39%, *n* = 226) or between three months and less than five months (23%, *n* = 136). Fewer sites reported never closing (18%, *n* = 105), closing between one day and less than one month (10%, *n* = 60), and closing for 5 months or longer (10%, *n* = 59). The reported site closure duration was largely the same across CACFP and non-CACFP participating sites with the exception of sites having never closed and sites closed for five months or longer. Approximately one quarter (24%, *n* = 62) of CACFP participating sites never closed, while 13% (*n* = 43) of non CACFP participating sites never closed. For sites closed for five months or longer, approximately 7% (*n* = 19) participated in CACFP and 12% (*n* = 40) did not participate in CACFP. The most frequently reported changes to service models due to the COVID-19 pandemic were reducing hours or days open (47%, *n* = 279), limiting the number of children served (46%, *n* = 274), and offering virtual education for children and families (43%, *n* = 257). CACFP and non-CACFP participating sites were similar in most reported changes in service models, but more non-CACFP participating sites limited the number of children served (50%, *n* = 168) compared to CACFP participating sites (41%, *n* = 104).

### 3.3. Approaches to Connecting Families to Food and Nutrition Resources

Overall, just under half of all respondents (48%, *n* = 281) reported no approaches to connecting families and children to food and nutrition resources amid the COVID-19 pandemic (Table 3). Of CACFP participating sites, 42% (*n* = 106) reported no approaches and 52% (*n* = 175) of non-CACFP participating sites reported no approaches. For sites reporting such approaches, the most frequently reported initiative was providing recommendations for community food resources (including food pantries and Supplemental Nutrition Assistance Program (SNAP)) (38%, *n* = 222). Less frequently reported approaches include offering “grab and go” (non-congregate meals) (12%, *n* = 68), distributing food boxes to families (11%, *n* = 67), offering meal delivery (6%, *n* = 35), and providing food from on-site gardens for families (4%, *n* = 23). These results were similar across sites participating in CACFP and sites not participating in CACFP, although a slightly larger proportion of sites participating in CACFP reported conducting each approach compared to sites not participating in CACFP. The mean total number of approaches used by sites that reported at least one approach was 1.5 (SD = 0.8) overall, 1.6 (SD = 0.9) for CACFP participating sites, and 1.4 (SD = 0.7) for non-CACFP participating sites.

### 3.4. Approaches to Connecting Families to Food Resources by CACFP Status

After adjusting for site enrollment and program type, CACFP participating sites were significantly more likely to offer “grab and go” (non-congregate) meals (OR = 3.9, 95% CI [2.1, 7.1]; *p* < 0.001), offer meal delivery (OR = 4.4, 95% CI [1.9, 10.0]; *p* < 0.001), distribute food boxes to families (OR = 2.6, 95% CI [1.5, 4.5]; *p* = 0.001), and to recommend community food resources (OR = 1.5, 95% CI [1.0, 2.1]; *p* = 0.04) than non-CACFP participating respondents (Table 4). CACFP participating sites were less likely to report using no approaches to connecting children and families to food during the COVID-19 pandemic (OR = 0.6, 95% CI [0.4, 0.8]; *p* = 0.003) (Table 5). Disaggregated by site closure, the difference was only significant for sites closed three months or longer (OR = 0.4, 95% CI [0.2, 0.7]; *p* = 0.002). The mean number of farm to ECE activities among respondents reporting at least one activity was not significantly different between CACFP and non-CACFP sites except for sites that closed 3 months or longer (IRR = 1.5, 95% CI [1.1, 2.0]; *p* = 0.02).

## 4. Discussion

This study presents results from a cross-sectional survey of 589 responding ECE sites from two U.S. states aimed at exploring approaches to providing food and nutrition resources for children and families during the COVID-19 pandemic. Of responding sites, 43% participated in CACFP and 57% did not participate in CACFP. Most sites, regardless of CACFP status, reported that they were “significantly” impacted by the COVID-19 pandemic and were most likely to have closed for between one month and less than three month due to the COVID-19 pandemic. When it comes to connecting families to food and nutrition resources, CACFP participating sites were more likely to report using any approaches and significantly more likely to report offering “grab and go” meals, providing meal delivery, distributing food boxes to families, and providing recommendations for community food resources. Among sites closed for greater than three months, the mean number of approaches to connecting families to food and nutrition resources was significantly higher in CACFP participating sites than non-CACFP participating sites. Non-CACFP sites were significantly more likely to report using no approaches to connect families to food and nutrition resources.

The primary findings confirm the researchers’ initial hypothesis that CACFP sites would be more likely to provide food and nutrition resources to families during the COVID-19 pandemic. Previous literature indicates that CACFP participating sites were more likely to provide family nutrition education and engagement than non-CACFP participating sites [15,35]. Non-CACFP sites may experience more barriers to communicating with parents about nutrition [15]. An existing precedent of communication with parents about food and nutrition may have better set the stage for CACFP participating sites to provide resources during the COVID-19 pandemic. Existing barriers to reaching parents reported in non-CACFP participating sites were likely exacerbated in the midst of the COVID-19 pandemic. In previous literature, Head Start sites, which are required by U.S. federal law to participate in CACFP, were even more likely to provide parents with nutrition education than non-Head Start CACFP sites and non-CACFP participating sites, potentially due to the Head Start policies that require family engagement and supplemental training provided to Head Start staff [35]. Further research is required to better understand how Head Start response may have differed from other program types and how existing nutrition and family engagement policy may have influenced food and nutrition practices amid the COVID-19 pandemic.

CACFP participating sites were also more likely to serve children and families with food insecurity than non-CACFP participating sites [16,36]. CACFP participating sites may have been responding to a greater need for continued food access experienced by the families they serve. CACFP has been shown to be an important potential pathway to supporting family food security by increasing the quality and quantity of food offered in the ECE setting and thus reducing food expenses for families [16,34]. As food insecurity rates increased suddenly and dramatically during the COVID-19 pandemic, especially for families with young children, this additional support likely became even more important for families [25]. Simply by continuing CACFP meal operations, sites that remained fully open to provide in-person care for children were providing a form of food resource to families. However, this study aimed to assess activities that went beyond providing meals as per usual. Initiatives that CACFP sites were significantly more likely to offer, including “grab and go” meals, family food boxes, and recommendations for community food resources, were likely aimed at supporting families who may have been experiencing new, ongoing, or deepened food insecurity. This may also account for the even greater significance of CACFP sites closed for three months or longer to provide food and nutrition supportive approaches, as the sites aimed to meet ongoing food security challenges. CACFP participating sites’ capacity to provide resources may be due to increased funds available through the CACFP reimbursement. Previous literature points to CACFP participating sites reporting fewer barriers to serving healthier foods and being less likely to report cost and lack of staff knowledge as barriers [20]. Without knowledge and cost as a barrier, CACFP participating sites may have had greater capacity to offer food and nutrition services and resources to families.

There were differences in site closure status between CACFP and non-CACFP participating sites, particularly across sites that never closed. This may suggest CACFP participating sites may have had more capacity to remain open to serve children and families. The approaches to connecting families to food and nutrition resources explored in this survey are applicable to sites that were open for in-person care, fully or partially, or just operational, that is they may have had staff working, but were not providing in-person care or learning. There is significant nuance and fluidity in this distinction because sites were frequently shifting closure status, changing capacity guidelines, and adapting to staffing issues and limitations. The study findings suggest that CACFP participating sites may have had more capacity to remain operational and offer services like “grab and go” meals and meal delivery that would be even more relevant during site closure. Because the survey did not ask specifically about periods of operation, additional research would be necessary to further explore this nuance.

Though CACFP sites were more likely to offer approaches to connecting families with food and nutrition resources, the relatively low number of CACFP sites that continued to offer meals through “grab and go” (16%, *n* = 42) or delivery (9%, *n* = 23) reflects the limited capacity for most ECE sites to continue to provide food and nutrition resources to families. While the USDA was granted ability to issue waivers for meal time flexibility and non-congregate feeding (allowing for “grab and go” and meal delivery) in CACFP programs through the Families First Coronavirus Response Act on March 18, 2020, delays in issuing the waivers and providing guidance for CACFP programs left many providers unsure if they would be reimbursed for meals and snacks served outside of the traditional CACFP model [29,37]. Overall CACFP program participation decreased dramatically throughout the COVID-19 pandemic [38]. From March through September of 2020, 480 million fewer CACFP meals were served than in the same time period in 2019, a 41% decrease [38]. According to Nutrition and Obesity Policy Research and Evaluation Network (NOPREN) COVID-19 Early Childcare and Education Working Group, the root of this breakdown in capacity for child care sites to continue to meet the nutritional needs of children and families is threefold: loss of income and reimbursement left child care sites unable to pay staff; child care providers, disproportionately within the “high-risk” category, closed sites or limited initiatives to protect their own health; and CACFP reimbursement is insufficient to cover food costs and administrative costs, let alone additional costs of transitioning service models [29].

Despite closures and limited capacity, ECE sites remained an important pathway to connect children and families to food resources. During the COVID-19 pandemic, food banks and emergency food assistance systems were severely strained. In many communities, food pantries could not meet the demand of the dramatically increased numbers of families experiencing food insecurity [39]. Additionally, families with young children were not included in some of the federal nutrition programs aimed at addressing childhood hunger during the COVID-19 pandemic. Specifically, children under six years of age were initially not eligible for Pandemic Electronic Benefits Transfer (P-EBT), a program that provides funding to families to purchase foods when children miss school meals due to school closure [40]. ECE sites must be one of a spectrum of resources to support families experiencing food insecurity. Based on existing relationships with caregivers and parents, ECE sites are especially well positioned to help families access and navigate other food assistance programs.

However, even outside of the COVID-19 pandemic, CACFP programming itself may not be reaching children and families that could be significantly benefitting from the program. Due to the eligibility limitations, lower-income children living in high-income areas may not have access to CACFP [36]. Lack of awareness, administrative burden and insufficient reimbursement may inhibit eligible ECE sites from choosing to participate in the program [11,34]. Additional research and qualitative exploration would provide deeper insight into the barriers that ECE providers faced in continuing to offer meals and food resources to children during the COVID-19 pandemic and how those barriers may or may not be alleviated with CACFP participation. For sites that are not eligible for CACFP, voluntary initiatives that prioritize nutrition and build relationships across families, community partners, and local food systems stakeholders can support continued access to food resources for families. This may include farm to child care initiatives that engage children and families or regularly assessing family food security and referring families to community food resources and programs [7]. While additional exploration is needed, state child care policies that include health and nutrition guidance may be one pathway to supporting healthier nutrition environments in ECE settings [7,41].

This study provides novel insight into the food and nutrition responses of ECE sites during the COVID-19 pandemic. However, it does have several limitations. First, the geographic scope of this study was limited to two U.S. states, Arizona and Pennsylvania. These states provide geographic and policy diversity, but it is not possible to generalize the results due to this limited geographic reach. Second, all site information is self-reported and not confirmed through any other sources. The individual completing the survey may not have been fully informed of all activities at a site and thus activities may have been underreported. Alternately, approaches to connecting families to food and nutrition resources may have been overreported due to perceived social desirability of those activities. Third, the low overall response rate (7.8%) may limit generalizability of survey results. Prior studies similar in nature have garnered response rates around 50% [11,42,43]. The low response rate in this survey is likely due to the impacts of the COVID-19 pandemic itself. When the survey was distributed (November 2020) there were still significant fluctuations in closure status for ECE sites and many ECE sites had already permanently closed but may not have yet been removed from official state lists. Sites that were opened experienced extremely limited staff capacity while also having to pivot program models and meet new health and safety protocols, which may have limited their capacity to respond to the survey. Future studies may consider telephone outreach and options to complete surveys verbally (via phone) or in written mail format to increase response rate. A final limitation is the lack of economic status information collected from survey respondents. The populations served by CACFP and non-CACFP participating sites could differ significantly in economic status and future studies should control for that difference.

## 5. Conclusions

No studies that the authors are aware of have examined ECEE sites approaches to providing families with food and nutrition resources during the COVID-19 pandemic by CACFP status. This study suggests that CACFP sites may have greater capacity to continue to connect families to food and nutrition resources in the midst of emergencies than non-CACFP participating sites. However, the small number of ECE providers, CACFP participating and non-participating, that continued to provide resources to families suggests that the CACFP program in its current form and ECE systems in general may be insufficient to fully support ECE sites in meeting the needs of families, particularly in a crisis. Further research is needed to better understand the barriers and facilitators to accessing and implementing CACFP and thus identify opportunities to increase reach of and participation in the program. Exploration of policy changes that would strengthen CACFP and its function as a pathway to increase food security for families is also needed. We are only now starting to see the long-term impacts of the COVID-19 pandemic, the ensuing economic crisis, and the federal response on the ECE system. Amid the COVID-19 pandemic, ECE sites emerged as an important connector to community food and nutrition resources for families. Proportionally fewer sites reported providing direct food resources, but for families able to access it, this was likely an especially vital source of food as the emergency food system was overwhelmed by demand. Though emergency funding and policy flexibilities sought to bolster ECE sites’ capacity to continue to serve children and families, longer-term investments and policy change may be required to rebuild ECE sites’ long-term resilience and capacities. With sufficient funding and capacity development, ECE sites can be one important entity in the network of resources ensuring comprehensive food security for children. The impacts of the COVID-19 pandemic on the ECE industry and the way that those impacts reverberate for the families of young children offer an opportunity to understand and elevate the current and potential role that ECE sites can play in supporting families and children in accessing food and nutrition resources.

## Figures and Tables

**Table 1:** Respondent site characteristics reported by CACFP participation status.

	Participating in CACFP	Not Participating in CACFP	Total
**Program Type ^a^**	**N (%)**		
Family or Group Child Care Home	83 (33)	44 (13)	127 (22)
Child Care Center	148 (59)	211 (63)	359 (61)
Head Start and/or Early Head Start	8 (3)	0 (0)	8 (1)
Preschool (state, private, or through a K-12 district)	13 (5)	78 (23)	91 (16)
**State**	**N (%)**		
Arizona	43 (17)	81 (24)	124 (21)
Pennsylvania	212 (83)	253 (76)	465 (79)
**Child Enrollment**	**Mean (SD)**		
February 2020	65 (86)	69 (92)	67 (90)
November 2020	43 (64)	45 (47)	44 (55)

^a^ Three centers participating in CACFP and one center not participating in CACFP did not indicate program type. CACFP = Child and Adult Care Food Program; N = number; SD = standard deviation.

**Table 2:** Reported impacts of COVID-19 on site function and service models by CACFP participation status.

	Participating in CACFP	Not Participating in CACFP	Total
**Impact of COVID-19 on Site ^a^**	**N (%)**		
None	3 (1)	6 (2)	9 (2)
Moderate	108 (43)	138 (41)	246 (42)
Significant	142 (56)	189 (57)	331 (56)
**Site Closure Duration ^b^**	**N (%)**		
Never closed	62 (24)	43 (13)	105 (18)
1 day to <1 month	30 (12)	30 (9)	60 (10)
Between 1 month and <3 months	91 (36)	135 (41)	226 (39)
Between 3 months and <5 months	52 (20)	84 (25)	136 (23)
5 months or longer	19 (7)	40 (12)	59 (10)
**Changes in service models due to COVID-19**	**N (%)**		
Offered virtual education for children and families	109 (43)	143 (43)	252 (43)
Reduced hours or days open	114 (45)	16 (49)	130 (22)
Limited services to children of essential workers only	45 (18)	53 (16)	98 (17)
Limited the number of children served	104 (41)	168 (50)	272 (46)

^a^ Two centers participating in CACFP and one center not participating in CACFP did not indicate impact of COVID-19 on site. ^b^ One center participating in CACFP and two centers not participating in CACFP did not indicate site closure duration. CACFP = Child and Adult Care Food Program; N = number.

**Table 3:** Reported approaches to connecting families to food and nutrition resources by CACFP participation status.

	Participating in CACFP	Not Participating in CACFP	Total
**No approaches to connecting families to food and nutrition resources**	106 (42)	175 (52)	281 (48)
**Specific approaches to connecting families to food and nutrition resources**	**N (%)**		
Offered “grab and go” meals	42 (16)	26 (8)	68 (12)
Offered meal delivery	23 (9)	12 (4)	35 (6)
Distributed food boxes	39 (15)	28 (8)	67 (11)
Provided food from on-site garden	14 (5)	9 (3)	23 (4)
Recommended community food resources	107 (42)	115 (34)	222 (38)
**Total number of approaches to connecting families to food and nutrition resources reported ^a^—Mean (SD)**	1.6 (0.9)	1.4 (0.7)	1.5 (0.8)

^a^ Among sites reporting at least one approach. CACFP = Child and Adult Care Food Program; N = number; SD = standard deviation.

**Table 4:** Unadjusted and adjusted ^a^ odds ratios for specific approaches reported by CACFP compared to non-CACFP sites ^b^.

Specific Approaches to Connecting Families to Food and Nutrition Resources	Unadjusted Odds Ratios (95% CI)	*p*-Value	Adjusted Odds Ratios (95% CI)	*p*-Value
Offered “grab and go” meals	2.6 (1.5, 4.4)	<0.001	3.9 (2.1, 7.1)	<0.001
Offered meal delivery	2.7 (1.3, 5.6)	0.007	4.4 (1.9, 10.0)	<0.001
Distributed food boxes	2.0 (1.2, 3.3)	0.01	2.6 (1.5, 4.5)	0.001
Recommended community food resources	1.4 (1.0, 1.9)	0.08	1.5 (1.0, 2.1)	0.04
Provided food from on-site garden	2.1 (0.9, 4.9)	0.10	1.6 (0.6, 3.9)	0.35

^a^ Adjusted for number of children enrolled and site type. ^b^ Among sites reporting at least one approach. CACFP = Child and Adult Care Food Program.

**Table 5:** Adjusted ^a^ odds ratios and incidence rate ratios for approaches connecting families to food and nutrition resources ^b^ reported by CACFP compared to non-CACFP sites, overall and by length of closure during the COVID-19 pandemic.

	**Odds Ratios (95% CI)**	***p*-Value**
No approaches to connecting families to food and nutrition resources ^b^	0.6 (0.4, 0.8)	0.003
Never closed (*n* = 105)	0.9 (0.4, 2.3)	0.87
Closed 1 day to <3 months (*n* = 286)	0.7 (0.4, 1.1)	0.13
Closed ≥3 months (*n* = 195)	0.4 (0.2, 0.7)	0.002
	**Incidence Rate Ratios (95% CI)**	***p*-Value**
Mean number of approaches connecting families to food and nutrition resources ^b^	1.2 (1.0, 1.5)	0.06
Never closed (*n* = 105)	0.7 (0.4, 1.3)	0.26
Closed 1 day to <3 months (*n* = 286)	1.2 (0.9, 1.5)	0.32
Closed ≥3 months (*n* = 195)	1.5 (1.1, 2.0)	0.02

^a^ Adjusted for number of children enrolled and site type. ^b^ Among sites reporting at least one approach. CACFP = Child and Adult Care Food Program; CI = confidence interval.
